# Coated recombinant target protein helps explore IL-1RAP CAR T-cell functionality in vitro

**DOI:** 10.1007/s12026-022-09348-y

**Published:** 2022-12-02

**Authors:** Mathieu Neto Da Rocha, Melanie Guiot, Clementine Nicod, Rim Trad, Lucie Bouquet, Rafik Haderbache, Walid Warda, Pierre-Emmanuel Baurand, Chloe Jouanneau, Philippe Dulieu, Marina Deschamps, Christophe Ferrand

**Affiliations:** 1 INSERM UMR1098 Right, Laboratoire de Thérapeutique Immuno-Moléculaire Et Cellulaire Des Cancers, EFS BFC, Univ. Bourgogne Franche-Comté, Jean François Xavier Girod, 8 Rue du Dr, 25000 Besançon, France; 2 Present Address: CanCell Therapeutics, 25000 Besançon, France; 3 Diaclone SAS, 25000 Besançon, France

## Background 

Many chimeric antigen receptor (CAR) T-cell therapies are being developed to treat various cancers. Five such therapies have been approved by regulatory agencies for blood cancers unresponsive to other treatments, such as B-cell acute lymphoid leukemia [1], B-lymphoma (diffuse large B-cell [2] and mantle cell lymphoma [3]), and multiple myeloma [4]. These therapies must be subjected to robust quality controls to ensure the safety of each batch and the final product. Considering high inter-donor or patient variability, a quality control strategy would help the Advanced Therapy Medicinal Product (ATMP) manufacturers to optimize and standardize their manufacturing processes, guaranteeing their reproducibility [5]. We need to quantify the potency of CAR T-cells using validated assays and good laboratory practices (GLP) before entering into pilot clinical trials (phase 3) to register for the ATMP.

The European Medicine Agency (EMA) defines potency as the measure of biological activity (target-specific cytotoxicity of CAR T-cells) using a bioassay, based on the attribute of the product (target antigen), which is linked to the relevant biological properties (cytotoxicity and related effects). Biological activity is the specific ability or capacity of a product to achieve a defined biological effect [6].

CAR T-cells activate when their extracellular domain comprising a single-chain fragment variable (scFv) antibody recognizes the target cell surface antigens. The signal travels through the transmembrane domain to the intracellular CD3-zeta costimulatory domain (mainly CD28- or 4-1BB-derived domains). This TCR-like activation induces proliferation, cytokine secretion, and cytotoxicity.

To date, measuring cytotoxicity is the preferred assay to assess CAR T-cell potency, although standardized assays are currently unavailable. The ^51^Cr-release assay remains the gold standard to assay cytotoxicity; however, it is hazardous and almost untransferable due to ^51^Cr radioactivity [7]. We need to develop alternative potency assays to quantify parameters such as transgene expression, proliferative capacities, phenotype (memory vs. effector), exhaustion phenotype, the release of lytic granules (perforin or granzyme A/B), or cytokine secretion that reflect CAR T-cell cytotoxicity.

Activated immune cells degranulate and release cytolytic enzymes. This process involves the fusion of the granule membrane with the cytoplasmic membrane of the immune effector cells, resulting in surface exposure of lysosomal-associated proteins present inside the lytic granules, such as CD107a glycoprotein-1 (LAMP1). Membrane expression of CD107a represents a surrogate marker of cytotoxicity of activated and degranulating immune cells. The relationship between CD107a expression and cytotoxicity has been well established in NK cells [8–10]. CD107a flow cytometry and microscopy are used to study the cytotoxicity of cytotoxic T lymphocytes (CTLs) [7, 11].

CAR T-cells can be activated using reagent-coated beads, target tumor cell lines, antigen-presenting cells (APC) [12], artificially engineered APC [13], or cell membrane-derived vesicles [14]. In this study, we developed a rapid, simple, efficient, and inexpensive assay to characterize our Interleukin-1 receptor accessory protein (IL-1RAP)-CAR T-cells [15]. We used a cell-free target, recombinant IL-1RAP (rIL-1RAP) protein coated on a substrate (96-well plates), to evaluate the cytotoxicity of IL-1RAP-CAR T-cells.

## Materials and methods

### Healthy donor blood samples and cell lines

We collected blood samples anonymously from healthy donors at the French Blood Center (Besançon, France) and isolated fresh peripheral blood mononuclear cells (PBMCs) using Ficoll Hypaque density centrifugation (Lymphocyte separation medium, Eurobio). The donors provided written informed consent, and the study was conducted according to the ethical guidelines (Declaration of Helsinki) and approved by the local CPP-Est (France).

We cultivated K562, CCL-243™, ATCC®), and Mono-Mac-6 myeloid (ACC-124™, DSMZ®, Germany) cell lines in complete medium (RPMI1640, 10% heat-inactivated fetal bovine serum, and 100 µM penicillin/streptomycin) at 37 °C with 5% CO_2_.

### Lentiviral construct, supernatant production, and ex vivo T-cell transduction

**Fig. 1:**
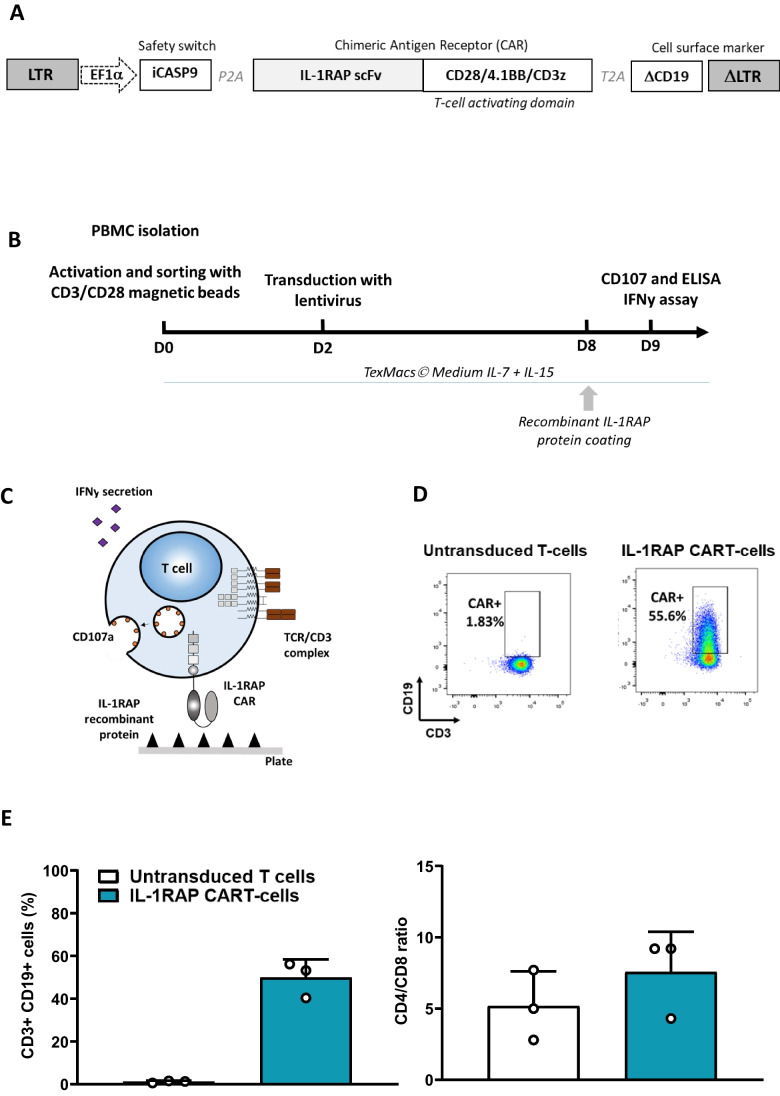
Schematic representation of lentiviral construct, IL-1RAP-CAR T-cell production and principle of coated recombinant target IL-1RAP-CAR T-cell recognition. **A** Schematic lentiviral construct crrying 3 different transgenes. **B** Workflow of IL-1RAP-CAR T-cell production and CD107a staining and IFNγ secretion quantification assays. **C** Schematic recognition of coated rIL-1RAP by IL-1RAP-CAR T-cells. **D** Representative cytometry plot of CD3 + /CD19 + staining of untransduced and IL-1RAP-CAR T-cells at day 9. **E Right**: Lentiviral transduction efficiency for donor T-cells measured using flow cytometry (*n* = 3). **Left**: CD4/CD8 T-cells ratio for cultured and untransduced and IL-1RAP-CAR T-cells. Results are presented as mean ± SD for 3 independent transductions of 3 different donor PBMCs

The CAR lentiviral construct (pSDY-iC9-IL1RAPCAR-∆CD19) (Fig. 1A) contains a procaspase-9 suicide gene safety switch, a ΔCD19 cell surface marker, and the IL-1RAP-CAR scFv sequence linked to two costimulatory domains (4-1BB and CD28) and one signaling domain (TCR-ζ). Lentiviral supernatant production has been previously described [15, 16].

We sorted healthy donor T-cells from PBMCs and activated them using CD3/CD28 microbeads (Dynabeads® CD3/CD28 CTS®, Gibco, Life Technologies). We transduced the T-cells on day 2 by adding a suspension of 10^5^ cells (without human serum (HS)) to each well containing 150 µl of concentrated IL-1RAP lentiviral supernatant (LvSN) in a 24-well plate. CAR T-cells were then expanded until day 9 in TexMacs™ medium (Miltenyi Biotec), supplemented with 350 IU/ml IL-7 and 56 IU/ml IL-15 (Miltenyi Biotec), 8% human serum (French Blood Center, Besançon, France), and 100 µM penicillin/streptomycin (Eurobio) at 37 °C and 5% CO_2_ (Fig. 1B).

The transduction efficiency was established by staining CD19 with an allophycocyanin (APC)–conjugated anti-CD19 antibody (clone LT19) and CD3 VioBlue (Clone REA613) (Miltenyi Biotec) and analyzing the transduced cell using flow cytometry (BD Canto II).

### IL-1RAP protein production and coating

The recombinant protein was developed and produced by Diaclone SAS (Besançon, France). The human IL-1RAP extracellular domain sequence ([Ser21 to Glu359], accession number: Q9NPH3.2) followed by a C-terminal Histag was cloned into an expression vector optimized for expression in mammalian cells. The cloning was validated by Sanger sequencing. The expression vector was transfected into CHO cells for 14 days. Purification was performed using immobilized metal affinity chromatography (IMAC) with nickel Sepharose resin.

The purified product was suspended in PBS (pH 7.4, 155 mM NaCl, 8 mM Na_2_HPO_4_, and 1.8 mM KH_2_PO_4_) and sterilized using 0.2 µm filters. The purity of the final product was evaluated using SDS-PAGE. We first validated our rIL-1RAP by comparing it with the rIL-1RAP from two other suppliers (Bio-Techne, Minneapolis, USA, and ACROBiosystems, Newark, USA) using ELISA with anti-IL-1RAP monoclonal antibodies (B-L43 and B-R58) (Diaclone, Besançon, France).

The IL-1RAP protein suspended in PBS (Gibco™, Thermofisher; France) was coated overnight at different concentrations (0.01 − 10 µg/ml) on the surface of a 96-well plate (Falcon® 24-well Clear Flat Bottom TC, Corning, France) (Fig. 1C).

### CD107a degranulation assay

We analyzed the results for CD107a staining with anti-CD107a-PE (BD Bioscience), anti-CD3-Pacific Blue (Miltenyi Biotec), anti-CD19-APC (Miltenyi Biotec), and anti-CD8 FITC (Diaclone) antibodies for each CAR T-cell using flow cytometry (BD Canto II, Becton Dickinson, le Pont de Claix, France). Nine days after production, we added CD107a-PE antibody to 10^5^ cells before stimulating the CAR T-cells with coated IL-1RAP protein or co-cultured living tumor cells (K562 and/or Mono-Mac-6) for 5 h at an effector:target (E:T) ratio of 1:5 in 96-well plates. We included a negative control (human serum albumin 4% (Albunorm™, Octopharma) or the medium alone (TexMACs™, 350 IU/ml IL-7, and 56 IU/ml IL-15)) for every experiment. The cultures were incubated for 1 h at 37 °C and 5% CO_2_, and for an additional 4 h in the presence of the secretion inhibitor monensin (BD GolgiStop™, BD Biosciences).

### IFNγ ELISA

Culture supernatants (from the CAR T-cell co-cultures with tumor cells or rIL-1RAP) were assessed for IFNγ secretion after 6 h of co-culturing (10^5^ CAR T-cells or T-cells at 0.5 × 10^6^ cells/ml) using the human IFNγ ELISA kit (Diaclone, Besançon, France) according to the manufacturer’s instructions.

### Statistical analysis

Graphical and statistical analyses were performed using GraphPad software 8.0.2 by ANOVA statistical test.

## Results

### Production of IL-1RAP-CAR T-cells

Our IL-1RAP-CAR LvSN transduced primary T-cells efficiently (49.93 ± 8.39%, *n* = 3, Fig. 1D and E) in an ex vivo production process (using IL-7 and IL-15 cytokines). The CD4/CD8 ratio in LvSN-transduced IL-1RAP-CAR T-cells is similar to that in the untransduced/cultured T-cells (7.56 ± 2.82% vs. 5.16 ± 2.45%, respectively, *n* = 3) (Fig. 1E).

### Coated rIL-1RAP stimulates IL-1RAP-CAR T-cells, inducing cytotoxic degranulation

**Fig. 2:**
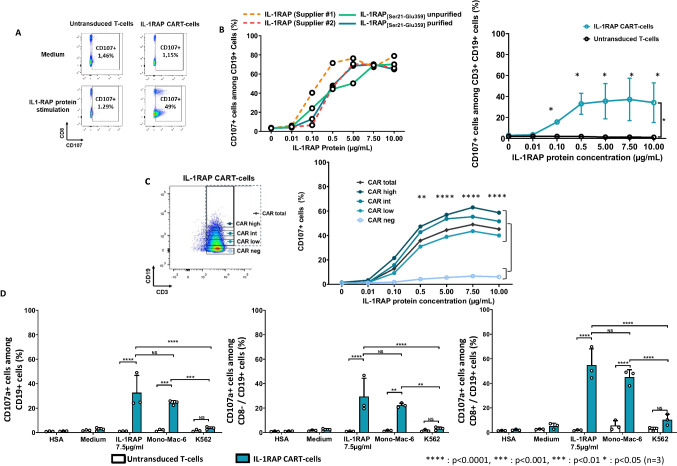
CD107a expression of IL-1RAP-CAR T-cells after stimulation with rIL-1RAP. **A** Representative flow cytometry analysis of CD107a degranulation assay. Untransduced T-cells and IL-1RAP-CAR T-cells were co-cultured (with or without the medium) with coated IL-1RAP protein for 6 h. CD107a^+^ cells staining was analyzed using flow cytometry gating on CD3^+^/CD19^+^ cells. **B Left**: Analysis of CD107a degranulation assay of CD19^+^ transduced T-cells after stimulation with different concentrations (0.01 up to 10 µg/ml) of rIL-1RAP from different suppliers (dotted lines) and our own rIL-1RAP in purified and unpurified forms (solid lines). **Right**: Percentage of total CD3^+^/CD19^+^/CD107a^+^ in untransduced or IL-1RAP-CAR T-cells co-cultured with different concentration of coated rIL-1RAP. Data are presented as mean ± SD for 3 independent experiments. **C Left**: Intensities of CD19 staining (negative, low, intermediate, or high) in transduced cell population (CD3^+^/CD19^+^) analyzed using flow cytometry. **Right**: Percentage of total CD3^+^/CD19^+^/CD107a^+^ or CD3^+^/CD19^−^/CD107a^+^ cells co-cultured with different concentration of coated rIL-1RAP. Data are presented as mean ± SD for 3 independent experiments. **D** CD107a degranulation assay for untransduced T-cells (white bars) and IL-1RAP-CAR T-cells (green bars) co-cultured at an E:T ratio of 1:5 for 6 h with target cells expressing or not expressing IL-1RAP (Mono-Mac-6 and K562, respectively) or co-cultured with coated rIL-1RAP (7.5 µg/ml). After 6 h, CD3^+^/CD19^+^/CD107a^+^ (left), CD3^+^/CD19^+^/CD8^−^/CD107a^+^ (middle), and CD3^+^/CD19^+^/CD8^+^/CD107a.^+^ (right) cells were analyzed using flow cytometry. Data are presented as mean ± SD for 3 independent experiments. ****p < 0.0001, ***p < 0.001, **p < 0.01, *p < 0.05

The purified rIL-1RAP was first validated at different coating concentrations (0, 0.01, 0.1, 0.5, 5, 7.5, and 10 µg/ml) for CD3 + /CD19 + (transduced cells) and compared with the recombinant proteins from two other suppliers using CD107a staining (Fig. 2A). The rIL-1RAP was comparable to the recombinant proteins from the suppliers at different purification stages (Fig. 2B (left)). We observed a significant dose–effect (*p* < 0.05, *n* = 3) at 0.01 µg/ml and a plateau at 5 to 10 µg/ml (Fig. 2B (right)). For quality control experiments (CD107a degranulation or IFNγ assays), we used a single concentration of purified rIL-1RAP (7.5 µg/ml).

To verify whether IL-1RAP-CAR was constructed successfully and degranulated upon recognizing the coated rIL-1RAP, we indirectly checked transgene expression using ΔCD19 cell surface staining. Both transgenes are separated by a T2A sequence that allowed stoichiometric protein expression. We thus analyzed the level of CD107a in different CD19 expressing subpopulations of transduced T-cells (Fig. 2C (left)) co-cultured with varying concentrations of coated rIL-1RAP. We noted a significant difference between CD107a staining of IL-1RAP-CAR T-cells and untransduced T-cells starting at 0.1 µg/ml of rIL-1RAP independent of the CD19 transgene expression level. We also observed a positive correlation between the CD107a expression and CD19 cell surface staining in CAR T-cells (Fig. 2C (right)).

Finally, we showed that CD107a cytotoxic degranulation of CAR T-cells and untransduced T-cells after co-culturing them with rIL-1RAP (36.22 ± 18.61 vs. 1.32 ± 0.26%, respectively, *n* = 3) was comparable to when they were co-cultured against living Mono-Mac-6 cell line (26.58 ± 3.44 vs. 4.01 ± 1.5%, respectively, *n* = 3) (Fig. 2D).

Control co-cultures with IL-1RAP^−^ K562 cell line or in the presence of albumin from human serum (HSA) did not induce cytotoxic degranulation. Gating of CD8^−^ (i.e., CD4^+^) or CD8^+^ subpopulations showed the same results.

### Induced IFNγ secretion by IL-1RAP-CAR T-cells after co-culture with coated rIL-1RAP

**Fig. 3:**
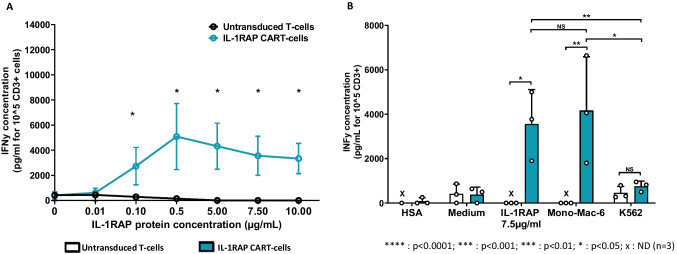
IFNγ secretion from IL-1RAP-CAR T-cells after stimulation with rIL-1RAP. **A** Quantification of IFNγ secretion in the supernatant of untransduced T-cells and IL-1RAP-CAR T-cells co-cultured with different concentrations of coated rIL-1RAP. Data are presented as mean ± SD for 3 independent experiments from 3 different donors. **B** Concentration of IFNγ (pg/ml) in the supernatant of untransduced T-cells and IL-1RAP-CAR T-cells co-cultured at an E:T ratio of 1:5 for 6 h with target cells expressing or not expressing rIL-1RAP (Mono-Mac-6 and K562, respectively) and co-cultured with coated rIL-1RAP. Data are presented as mean ± SD for 3 independent experiments. ****p < 0.0001, ***p < 0.001, **p < 0.01, *p < 0.05

We showed that the coated rIL-1RAP induced a maximum IFNγ secretion at a concentration of 0.1 µg/ml in CAR T-cells, which was significantly more than the IFNγ secretion by the untransduced T-cells (*p* > 0.05, *n* = 3), with a plateau at 0.5 µg/ml (Fig. 3A). The coated rIL-1RAP comparably assesses the CAR T-cell potency as a co-culture with living IL-1RAP cell surface antigen (undetectable vs. 3557.00 ± 1559.05 pg/ml and 756.66 ± 230.89 vs. 4166.66 ± 2420.3 pg/ml for IL-1RAP protein and the Mono-Mac6 line, respectively, *n* = 3) does. Specific IFNγ secretion was confirmed by the absence of secretion when cells were co-cultured with HSA or only the medium (Fig. 3B).

## Discussion

Assaying CAR T-cell functionality before using them clinically is essential to ensure the efficacy and safety of the drug. Implementing and standardizing such analytical assays can be difficult if cell lines are used, compromising their reproducibility. Thus, there is a need to develop a manageable, simpler, and easily reproducible method for antigen-specific stimulation of CAR T-cells. To explore CAR T-cell potency and cytotoxicity, well-characterized target cells (e.g., tumor cell lines) are used. However, cultures, especially multiple passages, may affect the health and behavior of the cells, affecting their transcriptomic activity and protein expression. We would require large-scale production and testing of cell lines, which would need to be cryopreserved. An aliquot would be defrosted each time we need to assay the CAR T-cells. Moreover, mistakes or an inversion of the cultured cell lines are unavoidable.

We hypothesized that a simple recognition interaction with the cognate antigen would stimulate CAR T-cells. Thus, we explored the use of a target antigen coated on plastic as an alternative to using cell lines expressing target antigen for studying CAR T-cell-specific stimulation and cytotoxicity. We demonstrated that the target of our third generation IL-1RAP-CAR T-cell-rIL-1RAP human protein coated on a plastic well plate—interacts and stimulates similarly the CAR T-cells just like the Mono-Mac-6 IL-1RAP^+^ cell line, which is known to stimulate the genetically modified IL-1RAP T-cells [16].

After engaging with the antigen, the T-cell receptor (TCR) forms an immune synapse to recruit and reorganize various membrane proteins [17] in a bullseye structure. As a result, the intracellular actors mobilize to restructure the cytoskeleton. This polarizes the endosomal compartment to form lytic granules and cytokine vesicles that activate the cytotoxic function [18]. In CAR T-cells, while the classical bullseye structure is maintained, the CAR-mediated synapses display a non-classical structure that is rapidly triggered after CAR-antigen interaction without the need for microtubule polarization. The CAR-mediated immune synapses induce cytotoxicity faster than the conventional TCR-mediated immune synapses [19, 20]. This explains why a coated protein target can stimulate a CAR T-cell without forming true physiologic immunologic synapse and can be used in an in vitro functional assay.

This technique could be helpful in transcriptomic, metabolic, or phenotypic studies of CAR T-cells. It limits the interference of nucleic acids present in target cell lines and analysis bias linked to the presence of residual tumor cells. It also avoids contamination by eliminating the need to sort CAR T-cells from the co-cultured cells [21]. Sorting cells using flow cytometry or purification columns can phenotypically alter or activate them.

In conclusion, with our IL-1RAP-CAR T-cells model, we demonstrated that a rIL-1RAP protein coated on a substrate could substitute cell lines as a target for CAR T-cells in cytotoxicity assays. This work demonstrates that this assay can be used for research practices and most importantly for QC delivery regarding potency of final CAR T-cell products, avoiding use of living cell line targets and allowing rapid results. However, the coated target protein needs to be validated at a GLP level before using it in clinical practice to test CAR T-cell potency.

## Data Availability

Raw datasets used during the current study are stored and available from the corresponding author on reasonable request.

## Abbreviations

APC: Antigen-presenting cells
ATMP: Advanced Therapy Medicinal Product
CAR: Chimeric antigen receptor
CTL: Cytotoxic T lymphocytes
EMA: European Medicine Agency
E:T: Effectors:targets
IFNγ: Interferonγ
LvSN: Lentiviral supernatant
PBMC: Peripheral blood mononuclear cells (PBMCs)
(r)IL-1RAP: (Recombinant)interleukin-1 receptor accessory protein
ScFv: Single-chain fragment variable
SD: Standard deviation
TCR: T-cell receptor
